# The Hidden Cost of Delay: Post-Pandemic Evolution of Advanced Ovarian Cancer Profiles

**DOI:** 10.3390/medicina62030598

**Published:** 2026-03-21

**Authors:** Alexandru Marius Petrusan, Catalin Vladut Ionut Feier, Calin Muntean, Vasile Gaborean, Andrei Stefan Petrusan, Delia Nicoara, Emil Marius Puscas, Florin Laurentiu Ignat, Patriciu Achimas-Cadariu

**Affiliations:** 1 Doctoral School, Iuliu Hațieganu University of Medicine and Pharmacy, 400012 Cluj-Napoca, Romania; petrusan.alexandru.marius@elearn.umfcluj.ro; 2 Department of Surgical Oncology and Gynecologic Oncology, Iuliu-Hatieganu Univeristy of Medicine and Pharmacy, 400012 Cluj-Napoca, Romania; mariusemilpuscas@gmail.com (E.M.P.); fignat2003@yahoo.com (F.L.I.); pachimas@umfcluj.ro (P.A.-C.); 3 Department of Surgical Oncology, Prof. Dr. I. Chiricuta Institute of Oncology, 400015 Cluj-Napoca, Romania; 4 Abdominal Surgery and Phlebology Research Center, Victor Babeș University of Medicine and Pharmacy, 300041 Timisoara, Romania; 5 First Surgery Clinic, “Pius Brinzeu” Clinical Emergency Hospital, 300723 Timişoara, Romania; 6 Medical Informatics and Biostatistics, Department III-Functional Sciences, Victor Babeş University of Medicine and Pharmacy Timişoara, Eftimie Murgu Square No. 2, 300041 Timişoara, Romania; 7 Thoracic Surgery Research Center, Victor Babeş University of Medicine and Pharmacy Timişoara, 300041 Timişoara, Romania; vasile.gaborean@umft.ro; 8 Department of Surgical Semiology, Victor Babeş University of Medicine and Pharmacy Timişoara, 300041 Timişoara, Romania; 9 Faculty of Medicine, Iuliu Hatieganu University of Medicine and Pharmacy, 400012 Cluj-Napoca, Romania; petrusan.andrei.stefan@elearn.umfcluj.ro (A.S.P.); delianicoara13@gmail.com (D.N.); 10 Department of Quality Management, Prof. Dr. I. Chiricuță Institute of Oncology, 400015 Cluj-Napoca, Romania

**Keywords:** high-grade serous ovarian carcinoma, COVID-19 pandemic, surgical outcomes, neoadjuvant chemotherapy, FIGO stage, postoperative recovery

## Abstract

Background and Objectives: High-grade serous ovarian carcinoma (HGSOC) remains the most lethal gynecologic malignancy, with outcomes heavily dependent on early diagnosis and timely multimodal treatment. The COVID-19 pandemic profoundly disrupted oncologic care, leading to diagnostic delays, modified treatment algorithms, and deferred surgeries. This study aimed to assess how these disruptions influenced disease presentation, surgical complexity, and postoperative outcomes during the pandemic and post-pandemic periods in a Romanian tertiary oncology center. Materials and Methods: A retrospective, single-center cohort analysis was conducted on 112 patients with histologically confirmed HGSOC who underwent surgical treatment between 26 February 2020 and 25 February 2024. The cohort was divided into two equal groups: a pandemic cohort (2020–2022) and a post-pandemic cohort (2022–2024). Clinical, pathological, and therapeutic parameters were compared, including FIGO and T staging, surgical duration, ICU admissions, and treatment intervals. Results: The post-pandemic period was marked by a significant rise in advanced-stage presentations (FIGO IV: 17.8% vs. 33.9%, *p* = 0.003), peritoneal carcinomatosis (58.9% vs. 82.1%, *p* = 0.004), and multiorgan invasion (7.1% vs. 16.0%, *p* = 0.039). Mean operative time increased significantly post-pandemic (94.0 ± 36.3 vs. 123.5 ± 52.5 min, *p* = 0.003), as did ICU admissions (35.7% vs. 60.7%, *p* = 0.002). While the number of neoadjuvant and adjuvant chemotherapy cycles remained consistent between cohorts, a greater surgical complexity and longer postoperative recovery characterized the post-pandemic cases, suggesting cumulative disease progression and increased treatment demands. Conclusions: The findings indicate an association between the post-pandemic period and more advanced disease profiles at presentation, as well as increased surgical complexity, highlighting potential long-term effects of healthcare disruption. These results highlight the necessity for resilient cancer care systems emphasizing early detection, multidisciplinary coordination, and adaptive treatment models to mitigate future systemic disruptions and preserve survival outcomes in women with HGSOC.

## 1. Introduction

Among gynecologic malignancies, epithelial ovarian cancer (EOC) remains one of the most aggressive and life-threatening entities. According to the World Health Organization (GLOBOCAN 2022), ovarian cancer accounts for more than 313,000 new cases and 207,000 deaths each year, ranking as the eighth leading cause of cancer-related mortality in women worldwide [1]. The high-grade serous ovarian carcinoma (HGSOC) subtype represents approximately 70–75% of all epithelial cases and is responsible for nearly 90% of ovarian cancer deaths [2,3]. Its high fatality rate primarily reflects delayed diagnosis at advanced stages and rapid biological progression, which together limit curative potential [4]. Despite major advances in surgical cytoreduction and systemic chemotherapy, the five-year overall survival for patients with stage III–IV disease remains below 40%, with recurrence commonly observed within the first two years after treatment completion [5].

Across Europe, ovarian cancer incidence and mortality display marked geographic variability, with higher rates reported in Central and Eastern Europe. In Romania, the age-standardized incidence is 12.3 cases per 100,000 women, exceeding both the overall European average (10.7 per 100,000) and the Western European average (8.2 per 100,000). Mortality follows a similar pattern, with an age-standardized rate of 7.4 per 100,000 in Romania compared to 6.0 across Europe and 4.7 in Western Europe. The mortality-to-incidence ratio is also higher in Romania (0.60) than in Western Europe (0.57), suggesting less favorable survival patterns [6]. This discrepancy is largely attributed to late-stage diagnosis, delays in treatment initiation, and limited access to advanced molecular testing, particularly BRCA1/2 and homologous recombination deficiency (HRD) screening, which restrict the use of personalized therapies [7].

Within the Romanian healthcare system, HGSOC is the most frequently encountered histological subtype and represents the main contributor to ovarian cancer–related mortality [8]. Most cases are treated in tertiary oncology centers, such as the “Ion Chiricuță” Oncology Institute in Cluj-Napoca, where multidisciplinary tumor boards and integrated surgical–chemotherapeutic approaches form the standard of care [7,8]. Nevertheless, regional disparities in resource allocation and delayed access to specialized oncology services continue to hinder early diagnosis and optimal treatment outcomes compared with Western European standards [6,7,8].

The recent global history has been profoundly shaped by the COVID-19 pandemic—an unprecedented event that caused severe and widespread disruptions across healthcare systems worldwide, with Romania being no exception. At the onset of 2020, the majority of healthcare resources were redirected toward managing COVID-19–positive patients, resulting in the temporary suspension or restriction of oncological services, with only emergency interventions permitted [9]. These system-wide adjustments led to delays in cancer diagnosis and treatment, ultimately contributing to disease progression and poorer prognoses among oncology patients [10,11].

For ovarian cancer, the impact was particularly significant, as treatment success relies heavily on timely diagnosis and strict adherence to multimodal therapeutic regimens, including cytoreductive surgery and chemotherapy [12]. Multiple reports have demonstrated that the pandemic period was associated with advanced stage at presentation, reduced surgical capacity, and altered chemotherapy scheduling, which collectively may have influenced short- and long-term outcomes [11,13].

During the COVID-19 pandemic, many oncology centers implemented modified versions of the European Society for Medical Oncology (ESMO) and Society of Gynecologic Oncology (SGO) treatment recommendations, frequently opting for neoadjuvant chemotherapy (NACT) instead of primary debulking surgery (PDS) to minimize perioperative risk and conserve healthcare resources. Although this shift was necessary at the time, evidence suggests it may have influenced disease evolution and patient outcomes, with higher proportions of advanced-stage diagnoses, increased rates of peritoneal carcinomatosis, and a greater need for intensive care unit (ICU) monitoring following surgery [11,14,15]. The wider use of NACT, while effective in reducing surgical morbidity, has also been associated with delayed cytoreductive interventions and potentially compromised survival outcomes in certain subgroups [16].

The post-pandemic period brought a different set of challenges. As hospital capacities normalized, oncology departments experienced a surge of patients whose treatments had been postponed during the health crisis. Many of these women presented with more extensive peritoneal spread, or multiorgan invasion at diagnosis, reflecting the cumulative effect of delayed care [17]. Recent analyses have identified a rebound phenomenon, characterized by a temporary rise in late-stage presentations even after restrictions were lifted, underscoring the long-term impact of disrupted care pathways [13,18].

The cornerstone of HGSOC management is complete cytoreductive surgery (R0) combined with platinum-based chemotherapy. Traditionally, patients with favorable performance status and resectable disease undergo primary debulking surgery (PDS) followed by adjuvant chemotherapy. When complete resection is deemed unlikely, neoadjuvant chemotherapy (NACT) followed by interval debulking surgery (IDS) serves as the established alternative standard of care [4,15].

Randomized trials and meta-analyses have demonstrated comparable overall survival outcomes between PDS and NACT–IDS when optimal cytoreduction is achieved, though PDS may yield superior long-term results in highly selected patients [4,15,16]. The therapeutic strategy is typically determined by preoperative imaging findings, CA-125 levels, comorbidities, and institutional surgical expertise. Moreover, the advent of PARP inhibitors and targeted maintenance therapies has significantly refined treatment paradigms, especially among patients with BRCA mutations or homologous recombination deficiency [16,19].

Nevertheless, in middle-income countries such as Romania, access to these novel therapies remains inconsistent, reinforcing the reliance on timely surgery and chemotherapy sequencing as primary determinants of survival.

Given the complexity of ovarian cancer management and the multifaceted disruptions caused by COVID-19, assessing how both the pandemic and the post-pandemic transition period influenced treatment patterns, surgical complexity, and postoperative recovery is essential. Through this analysis, the study contributes to a broader understanding of how major healthcare disruptions influence oncologic care continuity and highlights the importance of resilient, adaptable cancer management frameworks that can withstand systemic crises while maintaining treatment quality for patients with high-grade serous ovarian carcinoma.

## 2. Materials and Methods

This retrospective, observational, single-center cohort study aimed to evaluate patients who underwent total hysterectomy with bilateral salpingo-oophorectomy treatment for HGSOC at the “Ion Chiricuță” Oncology Institute in Cluj-Napoca, Romania. Patient data were retrospectively retrieved from institutional medical records and analyzed. The study included all women who underwent surgery for HGSOC between 26 February 2020, and 25 February 2024.

To assess the impact of the COVID-19 pandemic on the management and therapeutic outcomes of these patients, the observation period was divided into two distinct intervals: a pandemic cohort (26 February 2020–25 February 2022) and a post-pandemic cohort (26 February 2022–25 February 2024).

The selection of 26 February 2020, as the starting point of the pandemic cohort was based on the confirmation of the first COVID-19 case in Romania and the subsequent implementation of national public health restrictions, which directly affected hospital admission policies, elective surgical scheduling, and oncology service organization within our institution. The transition to the post-pandemic cohort (26 February 2022) corresponds to the period immediately preceding the official lifting of national COVID-19–related restrictions on 8 March 2022, marking the beginning of healthcare system normalization. Therefore, to comprehensively assess the pandemic’s influence on the surgical management of patients with high-grade serous ovarian carcinoma, the study period was structured into two consecutive and comparable two-year intervals representing the pandemic and post-pandemic phases.

For this study, specific inclusion criteria were established to ensure the homogeneity and reliability of the analyzed cohort. Eligible participants met all of the following conditions:

• Histopathological confirmation of high-grade serous ovarian carcinoma (HGSOC) following surgery;
• Completion of the entire multidisciplinary treatment protocol (surgery and chemotherapy) exclusively at the Ion Chiricuță Oncology Institute (IOCN);
• Surgical treatment performed within the defined study period (26 February 2020–25 February 2024);
• Availability of a pre-treatment pelvic MRI;
• Availability of a thoraco-abdominal CT scan prior to initiation of therapy;
• Treatment decision made by the institutional multidisciplinary tumor board;
• No documented history of SARS-CoV-2 infection prior to or during hospitalization;
• Negative RT-PCR test for SARS-CoV-2 upon admission;
• Availability of BRCA mutation testing results.

It is well established that active SARS-CoV-2 infection significantly worsens the postoperative course and overall prognosis of patients with malignancies, including those with gynecologic cancers [20,21]. Since the present study focuses on the indirect impact of the COVID-19 pandemic on the management of patients with HGSOC, and not on the direct consequences of SARS-CoV-2 infection itself, individuals with a documented history of COVID-19 prior to surgery or during hospitalization were excluded from the analysis.

Once all inclusion criteria were fulfilled, a total of 112 patients were enrolled in the study cohort, with 56 cases assigned to each study period. Multiple clinical and pathological variables were analyzed, including demographic characteristics (age and place of residence—urban vs. rural), and the presence of comorbidities, assessed using the Charlson Comorbidity Index. Imaging-derived parameters were also examined, such as tumor T stage, the presence of peritoneal carcinomatosis, and BRCA mutation status. In addition, FIGO stage and the presence and extent of tumor invasion into adjacent organs were evaluated as key indicators of disease progression and surgical complexity. A standardized Enhanced Recovery After Surgery (ERAS) protocol was implemented at our institution and consistently applied throughout the entire study period, with no major modifications between the two cohorts.

Tumor invasion into adjacent organs was evaluated at two levels of analysis. First, an overall “organ invasion” variable was defined as a binary parameter (presence vs. absence of invasion), irrespective of the specific organ involved. Multiorgan invasion was defined as the involvement of two or more adjacent organs. Second, individual organ involvement was analyzed separately for sites with more than five cases (rectum, urinary bladder, sigmoid colon, and ureter). Less frequent invasion sites (fewer than five cases per organ), including the fallopian tube, cecum, small intestine, and iliac vessels, were grouped under “other organs” for descriptive purposes only and were not analyzed individually due to limited statistical power. Finally, the necessity for stoma creation during surgery was also analyzed as an indicator of disease extent and surgical aggressiveness.

The therapeutic approach was analyzed in detail, including the use of NACT and the number of administered cycles, as well as the number of ACT cycles received postoperatively. Additional perioperative parameters were evaluated, such as operative time, the need for postoperative intensive care unit (ICU) admission, length of ICU stay, and the requirement for postoperative red blood cell transfusions. Furthermore, the presence of disease progression following treatment was recorded.

Finally, the study assessed several treatment interval durations, including:

• Days between radiologic diagnosis and surgical intervention;
• Time in days between histopathological diagnosis and initiation of NACT;
• Time in days between surgery and initiation of ACT;
• Postoperative length of hospital stay.

The study protocol was reviewed and approved by the Ethics Committee of the “Ion Chiricuță” Oncology Institute, Cluj-Napoca (approval no. 12252/18 December 2025). All procedures were conducted in accordance with the principles outlined in the Declaration of Helsinki and relevant national ethical regulations governing biomedical research.

### Statistical Analysis

All statistical analyses were conducted using IBM SPSS Statistics, version 25.0 (IBM Corp., Armonk, NY, USA). A two-tailed *p*-value < 0.05 was considered statistically significant. Continuous variables were expressed as mean ± standard deviation (SD), whereas categorical variables were summarized as absolute frequencies and percentages (%).

The normality of continuous data distributions was evaluated using the Shapiro–Wilk test. Depending on the results, either parametric or non-parametric tests were applied. Comparisons between two independent groups were performed using Student’s *t*-test for normally distributed data or the Mann–Whitney U (and, when appropriate, Kruskal–Wallis) test for non-normal distributions.

Categorical variables were analyzed using the Chi-square test, or Fisher’s exact test when expected frequencies were below five. Continuous variables were not categorized unless clinically justified. Correlations between continuous parameters were evaluated using Pearson’s correlation coefficient for parametric data and Spearman’s rank correlation for non-parametric data. Missing data were handled through listwise deletion, with no imputation procedures applied. In all analyses, a *p*-value of <0.05 was considered statistically significant, denoting that the observed differences were unlikely to be due to random variation.

## 3. Results

For this study, the medical records of patients who underwent surgery for high-grade serous ovarian carcinoma were reviewed. The analysis included cases operated between 26 February 2020, and 25 February 2022, representing the pandemic period. These data were subsequently compared with those from the post-COVID interval, 26 February 2022, to 25 February 2024, to evaluate temporal differences in clinical and surgical outcomes.

### 3.1. Patient and Disease Characteristics

Accordingly, data from 112 patients who met the inclusion criteria were analyzed. The mean age of the cohort was 57.2 ± 9.25 years, with 69 patients (61.6%) residing in urban areas. Additionally, 61 patients (54.46%) presented with at least one comorbidity besides HGSOC. The variation in demographic characteristics between the pandemic and post-pandemic periods is summarized in Table 1.

A statistically significant association was identified between age and organ invasion (*p* = 0.022), with patients exhibiting invasion having a higher mean age compared to those without invasion (59.15 ± 10.1 vs. 55.10 ± 8.7 years). A similar trend was noted for peritoneal carcinomatosis, where affected patients were older on average (58.44 ± 9.66 vs. 52.95 ± 7.5 years; *p* = 0.001).

Stoma formation was necessary in 5 cases (4.46%), all recorded in the post-pandemic cohort, and each case involved the creation of a colostomy.

Regarding BRCA gene status, positivity was reported in 23 cases (41.1%) during the pandemic and 19 cases (33.9%) in the post-pandemic period, with no statistically significant difference between the two proportions (*p* = 0.345).

While T stage variation was not significant, a statistically meaningful difference was observed in FIGO stage distribution between the two time intervals. Detailed results are presented in Table 2.

For T3 stage, an increase was observed from 74.9% in the pandemic to 85.7% in the post-pandemic period, although this difference was not statistically significant (*p* = 0.235). In contrast, the proportion of FIGO stage IV cases rose markedly post-pandemic (10 patients, 17.8% vs. 19 patients, 33.9%; *p* = 0.003).

A similar pattern emerged for peritoneal carcinomatosis, which increased significantly from 58.9% during the pandemic to 82.14% post-pandemic (*p* = 0.004).

Analysis of tumor invasion into adjacent organs revealed a non-significant increase in overall invasion frequency (25% vs. 33.9%; *p* = 0.196); however, the proportion of patients with multiorgan (≥2 organs) invasion showed a significant rise in the post-pandemic period (7.11% vs. 16.07%; *p* = 0.039).

Table 3 details the distribution of organ invasion across the two study intervals.

It is noteworthy that the invasion of additional structures such as the fallopian tube, iliac vessels, cecum, and intestinal loops was also observed, totaling six cases (two during the pandemic and four in the post-pandemic period). However, since the frequency for each individual site was fewer than five cases, no statistical analysis was performed for these variables.

### 3.2. Treatment Approach and Surgical Parameters

Of the total cohort, 61 patients (54.44%) received neoadjuvant chemotherapy. Although there was an approximate 10% increase in the use of this approach during the pandemic, the difference between the two periods was not statistically significant (33 patients, 58.9% vs. 28 patients, 50%; *p* = 0.337). The mean number of neoadjuvant chemotherapy cycles also remained comparable (6.5 ± 2.77 vs. 6.54 ± 3.4; *p* = 0.516). The remaining 51 patients (45.53%) underwent primary debulking surgery.

Regarding adjuvant chemotherapy, it was administered in 93 patients (83%), with no significant difference between the pandemic and post-pandemic groups (43 patients, 76.68% vs. 50 patients, 89.28%; *p* = 0.518). The mean number of adjuvant cycles was also similar across both intervals (10.12 ± 5.8 vs. 11.10 ± 6.67; *p* = 0.425).

In five patients (4.47%), the surgical procedure required the creation of a colostomy, all performed in the post-pandemic period (three FIGO IIIC, one IIIB, and one IVA case).

Disease progression occurred in eight cases (7.14%), with a higher frequency post-pandemic (5 patients, 8.9% vs. 3 patients, 5.3%; *p* = 0.247).

The mean operative time was significantly longer in the post-pandemic period (123.50 ± 52.51 min) compared to the pandemic (94.02 ± 36.28 min; *p* = 0.003).

Differences between the two periods regarding postoperative hospital stay, ICU admission and duration, and transfusion requirements are summarized in Table 4.

### 3.3. Treatment Timelines

Across the entire cohort, no statistically significant differences were observed in the waiting times between treatment stages. The detailed results are presented in Table 5.

However, as the two therapeutic strategies NACT and primary debulking surgery—follow distinct clinical pathways, a separate analysis was conducted based on the administration of NACT.

The variation in waiting times among the 61 patients who received NACT is presented in Table 6.

The variation in waiting times among the 51 patients who underwent primary debulking surgery is presented in Table 7.

## 4. Discussion

### 4.1. Disease Burden and Clinical Presentation

The COVID-19 pandemic was one of the most disruptive events in recent medical history, profoundly affecting healthcare systems worldwide. Even countries with strong financial and infrastructural capacity, such as the United States, Germany, and Japan, were overwhelmed and forced to implement emergency restrictions to manage the surge of infected patients [22,23,24]. Beyond the direct morbidity and mortality caused by SARS-CoV-2 infection, the pandemic led to significant alterations in oncologic care delivery. Elective cancer surgeries were widely postponed, resulting in delayed diagnoses and the presentation of patients at more advanced disease stages. Consequently, oncologic surgeries became more extensive, with higher rates of postoperative complications, longer ICU stays, and increased healthcare costs [9,10,11,18].

The psychological and emotional burden on patients was also considerable. Reduced access to healthcare facilities, fear of in-hospital infection, and public advisories discouraging non-urgent hospital visits led many patients—particularly those with HGSOC to defer consultations and diagnostic imaging [20,25]. This avoidance behavior contributed to a decline in early detection and screening, further shifting the disease spectrum toward late-stage diagnoses. The elderly and patients with comorbidities were disproportionately affected, often choosing to postpone both diagnostic investigations and the initiation of treatment due to infection-related anxiety [9,26].

In Romania, strict lockdown measures and limited hospital capacity accentuated these global trends. Oncologic institutions such as the Ion Chiricuță Oncology Institute experienced delays in patient triage, reductions in elective surgical volume, and increased rates of advanced-stage presentations at diagnosis [21]. As a result, the post-pandemic period was characterized by a rebound influx of cases presenting with multiorgan invasion, prolonged hospitalizations, and consequently poorer prognostic indicators in the medium and long term [13,18].

Our analysis revealed a significant post-pandemic rise in advanced-stage diagnoses (FIGO IV), increased peritoneal carcinomatosis, and higher rates of multi-organ invasion. These findings are consistent with reports from international cohorts showing stage migration toward advanced disease during the pandemic. Studies documented a 56% reduction in early-stage ovarian cancer diagnoses in São Paulo, Brazil, attributable to delays in screening and surgical restrictions [27], while similar trends were reported in India, emphasizing prolonged diagnostic intervals and treatment delays [28].

Across Europe, similar patterns were observed, with reports indicating a greater tumor burden at presentation and an increased proportion of complex cytoreductive surgeries requiring bowel or bladder resections during the COVID-19 pandemic. These findings reflect the delayed referrals and diagnostic bottlenecks that characterized oncologic care throughout the continent [6,10]. In our cohort, the proportion of patients with invasion of ≥2 organs nearly doubled post-pandemic, reflecting cumulative diagnostic delays and decreased outpatient surveillance.

Several mechanisms likely underlie this stage shift: limited outpatient visits, reduced radiologic capacity, patient fear of hospital exposure, and systemic triage policies prioritizing COVID-positive cases [9,29]. Moreover, emotional and socioeconomic stressors further discouraged women from seeking care, particularly older patients with comorbidities. These behavioral and structural factors explain the sustained presence of advanced-stage disease even after restrictions were lifted, confirming that the pandemic’s oncologic impact extended well beyond the acute phase [25,30].

### 4.2. Surgical and Therapeutic Adaptations

Our findings demonstrated a significant increase in operative duration and postoperative ICU admissions during the post-pandemic period. Importantly, these differences should be interpreted in the context of the substantially higher tumor burden documented in this cohort. The post-pandemic group exhibited a markedly greater proportion of FIGO stage IV cases, increased rates of peritoneal carcinomatosis, and a higher frequency of multiorgan invasion. Such disease characteristics inherently necessitate more extensive cytoreductive procedures, often involving complex resections and multivisceral surgical approaches. It is therefore clinically plausible that the observed prolongation of operative time and greater need for postoperative intensive care reflect the increased anatomical extent of disease rather than external system-level constraints. This interpretation aligns with established surgical principles in advanced ovarian cancer, where tumor dissemination and organ involvement are directly associated with procedural complexity and perioperative resource utilization. This pattern is consistent with data from multicenter French and European cohorts, where prolonged surgical times, increased blood loss, and extended ICU stays reflected greater tumor burden and strained surgical capacity during the pandemic [31,32].

Although the overall complication rate did not significantly change in our cohort, longer hospitalization (8.6 ± 5.4 days vs. 6.8 ± 1.6 days, *p* = 0.024) and ICU use (60.7% vs. 35.7%, *p* = 0.002) illustrate the pandemic’s enduring effect on perioperative management. These data correspond with international studies who observed increased postoperative morbidity and resource utilization despite similar chemotherapy completion rates [10,18,20]. The extension of postoperative recovery times likely resulted from both surgical complexity and delayed patient turnover due to persistent capacity constraints [33].

During the COVID-19 pandemic, treatment paradigms in ovarian cancer shifted markedly toward NACT, reflecting the ESMO and SGO recommendations to prioritize systemic therapy and reduce perioperative exposure. In our cohort, NACT was administered in 54.4% of cases, consistent with international data showing a temporary increase in NACT use across major oncologic networks, including Kaiser Permanente and the U.S. National Cancer Database, between 2020 and 2021, with a return to pre-pandemic levels thereafter [11,16,18]. Our institution’s pattern fits within this trajectory, reflecting adaptive but consistent adherence to evidence-based standards.

The observed increase in treatment intervals during the post-pandemic period highlights persistent disruptions in oncologic workflow even after the lifting of restrictions. In patients receiving neoadjuvant chemotherapy, the delay from surgery to adjuvant chemotherapy (49.5 vs. 36.3 days, *p* = 0.048) suggests residual system strain, consistent with international reports of chemotherapy initiation lag due to reduced hospital capacity and backlog accumulation [11,16].

Among patients selected for primary debulking surgery, the interval from radiologic diagnosis to operative intervention was significantly longer in the post-pandemic cohort (26.9 vs. 14.1 days, *p* = 0.028). This difference should be interpreted within the broader context of healthcare reorganization following the COVID-19 crisis. Although formal restrictions had been lifted, surgical services continued to operate under adjusted scheduling systems and accumulated case backlogs, which may have influenced preoperative timing. Considered together with the higher prevalence of advanced-stage disease in the same period, the prolonged interval suggests that the repercussions of pandemic-related disruption persisted beyond the emergency phase. While causality cannot be established, the temporal overlap between delayed surgical management and more extensive disease presentation indicates that the effects of the pandemic likely extended into the recovery phase of oncologic care delivery. This mirrors findings from multicenter cohorts in Italy and Finland, where preoperative scheduling delays persisted post-COVID owing to staff shortages and surgical triage limitations [10,13].

Furthermore, the rise in colostomy formation (4.5%, all post-pandemic) and multi-organ resections indicates that more patients presented with locally invasive disease requiring extensive surgery. This trend reflects the global disparities in access to optimal cytoreductive surgery that were magnified during the pandemic, as operating room restrictions and delayed oncologic referrals disrupted surgical care continuity [34,35].

### 4.3. Health System, Molecular Insights, and Future Directions

The impact of the COVID-19 pandemic on the treatment and management of HGOSC is undeniable. The continued presentation of patients with predominantly advanced-stage disease in the post-pandemic period underscores the long-term disruption of oncologic care and delays in diagnosis and therapy. Recent evidence indicates that even within the first two years following the resumption of full hospital activity, the proportion of advanced cases has remained markedly elevated [18,35].

In our cohort, the proportion of BRCA-positive patients remained comparable between the two periods, despite the marked increase in advanced-stage presentations after the pandemic. This observation aligns with reports showing that the decline in early gynecologic cancer diagnoses during COVID-19 was largely driven by reduced clinical access and delayed referrals rather than by interruptions in molecular testing capacity [36,37]. Large database analyses have similarly described stage migration without a corresponding reduction in biomarker assessment [18]. Together, these findings suggest that the main disruption occurred at the level of timely clinical evaluation, rather than within the molecular diagnostic framework itself [38]. Maintenance therapy with PARP inhibitors continued to evolve, with studies emphasizing strategic sequencing to optimize survival outcomes in the post-COVID era [39].

Our analysis underscores a paradox of recovery: while gynecologic oncology services have regained their procedural volume, the clinical quality of outcomes has yet to rebound. The post-pandemic patient now often arrives later, sicker, and harder to treat—a consequence of diagnostic inertia and care fragmentation lingering beyond the crisis [36]. These qualitative scars, marked by higher tumor burden and slower functional recovery, reveal that system restoration does not necessarily equate to system resilience [40].

Comparative analyses reveal a striking asymmetry in the pandemic’s aftermath: its impact was not evenly borne across healthcare systems. High-volume oncologic centers, fortified by specialized infrastructure and adaptive care protocols, largely preserved treatment continuity and mitigated excess mortality [41]. In contrast, low-volume or resource-limited hospitals endured disproportionate declines in surgical quality and survival outcomes, exposing the structural inequities that the pandemic merely amplified [37].

Future mitigation strategies must transcend mere crisis management, embedding adaptability into the architecture of oncologic care. Evidence underscores the value of flexible scheduling systems, tele-oncology integration, and algorithmic prioritization to safeguard elective cancer surgery during disruptions [42]. Emerging models advocate hybrid frameworks—combining virtual and in-person engagement—to sustain therapeutic continuity and patient trust across healthcare shocks [43]. As both our data and global experience affirm, revitalizing diagnostic vigilance and reinforcing multidisciplinary collaboration remain essential to improving survival for women with HGSOC in the post-pandemic era.

### 4.4. Study Limitations

This study presents several inherent limitations that should be acknowledged when interpreting the results. First, its retrospective and single-center design limits the generalizability of findings, as patient selection, treatment protocols, and institutional practices may differ across oncology centers. The data were obtained exclusively from the “Ion Chiricuță” Oncology Institute, a tertiary referral center, which could introduce a referral bias, favoring more advanced or complex cases. Second, the surgical procedures were performed by multiple surgeons, potentially introducing inter-operator variability in both the extent of cytoreduction and perioperative management. Similarly, histopathological examinations were conducted by different pathologists, each employing slightly distinct interpretative nuances. Third, radiologic investigations were interpreted by several radiologists, creating potential heterogeneity in measurement and preoperative staging accuracy. Moreover, the absence of a pre-pandemic control cohort limits the ability to fully disentangle pandemic-related effects from potential secular trends in disease presentation. Unmeasured confounders, including socioeconomic factors or evolving referral patterns, may have contributed to the observed stage distribution independently of the pandemic period. Therefore, the temporal associations identified in this study should be interpreted with caution and not as definitive causal inferences. Finally, the retrospective nature of data collection limited access to certain clinical details and patient-reported outcomes, emphasizing the need for prospective, multicenter studies to validate and expand upon these findings.

## 5. Conclusions

In conclusion, this study underscores the profound and lingering effects of the COVID-19 pandemic on the management of high-grade serous ovarian carcinoma. Despite the restoration of full hospital capacity, the post-pandemic landscape remains characterized by delayed presentation, advanced disease stages, and greater surgical complexity, reflecting the long-term erosion of diagnostic and therapeutic continuity. The observed increase in FIGO IV cases, multiorgan invasion, and ICU admissions highlights the cumulative toll of disrupted care pathways rather than transient system strain. Yet, these challenges also revealed the adaptability of multidisciplinary oncology teams, capable of sustaining core treatment quality through dynamic reorganization. Strengthening diagnostic vigilance, expanding molecular profiling, and integrating flexible, hybrid models of oncologic care will be pivotal in safeguarding outcomes for women with HGSOC against future healthcare disruptions.

## Figures and Tables

**Table 1:** Demographic aspects.

Variables	All (*n* = 112)	Pandemic (*n* = 56)	Post-Pandemic (*n* = 56)	*p*
Age (years, M ± SD)	57.2 ± 9.25	56.14 ± 9.65	58.27 ± 8.85	0.227
Environment				
Urban	69 (61.6%)	31 (55.4%)	38 (67.9%)	0.125
Rural	43 (38.4%)	25 (44.6%)	18 (32.1%)	
CCI > 3	62 (55.35%)	29 (51.7%)	33 (58.9%)	0.507

M = mean; SD = Standard Deviation; CCI = Charlson Comorbidity Index;.

**Table 2:** T stage and FIGO classification.

Variables	All (n = 112)	Pandemic (n = 56)	Post-Pandemic (n = 56)	*p*
T1a	4 (3.6%)	2 (3.6%)	2 (3.6%)	0.210
T1b	4 (3.6%)	4 (7.1%)	0	
T1c	5 (4.5%)	3 (5.4%)	2 (3.6%)	
T2a	6 (5.4%)	4 (7.1%)	2 (3.6%)	
T2b	3 (2.7%)	1 (1.8%)	2 (3.6%)	
T3a	8 (7.1%)	6 (10.7%)	2 (3.6%)	
T3b	26 (23.2%)	14 (25%)	12 (21.4%)	
T3c	56 (50%)	22 (39.2%)	34 (60.7%)	
FIGO Stage				0.048
I B	4 (3.6%)	4 (7.1%)	0	
I C	3 (2.7%)	2 (3.6%)	1 (1.8%)	
II A	4 (3.6%)	4 (7.1%)	0	
II B	1 (0.9%)	0	1 (1.8%)	
III A1	6 (5.4%)	2 (3.6%)	4 (7.1%)	
III A2	3 (2.7%)	3 (5.4%)	0	
III B	16 (14.3%)	8 (14.3%)	8 (14.3%)	
III C	46 (41.1%)	23 (41.1%)	23 (41.1%)	
IV A	12 (10.7%)	6 (10.7%)	6 (10.7%)	
IV B	17 (15.7%)	4 (7.1%)	13 (23.2%)	

**Table 3:** Organ invasion.

Variables	All	Pandemic	Post-Pandemic	*p*
Rectal invasion	13 (11.6%)	7 (12.5%)	6 (10.71%)	0.767
Bladder invasion	11 (9.8%)	4 (7.14%)	7 (12.5%)	0.230
Sigmoid invasion	10 (8.9%)	2 (3.5%)	8 (14.28%)	0.046
Ureteral invasion	6 (5.3%)	2 (3.5%)	4 (7.1%)	0.202

**Table 4:** Hospitalization aspects.

Variables	All (n = 112)	Pandemic (n = 56)	Post-Pandemic (n = 56)	*p*
Postoperative hospitalization (days, M ± SD)	7.68 ± 4.06	6.78 ± 1.62	8.61 ± 5.44	0.024
Postoperative ICU Yes	54 (48.2%)	20 (35.7%)	34 (60.7%)	0.002
Postoperative ICU (days, M ± SD)	2.84 ± 1.77	1.87 ± 1.24	3.42 ± 1.87	0.031
Transfusions Yes	24 (21.1%)	11 (19.64%)	13 (23.21%)	0.526

M = mean; SD = Standard Deviation.

**Table 5:** Critical timelines.

Interval	All (n = 112)	Pandemic (n = 56)	Post-Pandemic (n = 56)	*p*
Histopathological diagnosis to NACT (days, M ± SD)	27.26 ± 19.62	27.3 ± 21.11	27.21 ± 18.11	0.958
Radiologic diagnosis to surgery (days, M ± SD)	101.63 ± 73.98	107.16 ± 76.82	96.56 ± 71.87	0.988
Surgery to ACT (days, M ± SD)	41.42 ± 20.43	38.07 ± 11.84	44.44 ± 15.6	0.130

M = mean; SD = Standard Deviation.

**Table 6:** Critical timelines in NACT patients.

Interval	All (n = 61)	Pandemic (n = 33)	Post-Pandemic (n = 28)	*p*
Histopathological diagnosis to NACT (days, M ± SD)	27.26 ± 19.62	27.3 ± 21.11	27.21 ± 18.11	0.958
Radiologic diagnosis to surgery (days, M ± SD)	171.92 ± 47.57	171.58 ± 48.73	172.3 ± 47.32	0.988
Surgery to ACT (days, M ± SD)	42.73 ± 24.02	36.28 ± 9.54	49.46 ± 21.88	0.048

M = mean; SD = Standard Deviation.

**Table 7:** Critical timelines in debulking patients.

Interval	All (n = 51)	Pandemic (n = 22)	Post-Pandemic (n = 29)	*p*
Radiologic diagnosis to surgery (days, M ± SD)	21.53 ± 9.03	14.11 ± 7.84	26.88 ± 12.78	0.028
Surgery to ACT (days, M ± SD)	40.02 ± 15.9	40.3 ± 14.15	39.81 ± 17.4	0.918

M = mean; SD = Standard Deviation.

## Data Availability

The original contributions presented in the study are included in the article, further inquiries can be directed to the corresponding author.
